# IL-3 and TNFα increase Thymic Stromal Lymphopoietin Receptor (TSLPR) expression on eosinophils and enhance TSLP-stimulated degranulation

**DOI:** 10.1186/1476-7961-10-8

**Published:** 2012-07-28

**Authors:** Ellen B Cook, James L Stahl, Elizabeth A Schwantes, Kristen E Fox, Sameer K Mathur

**Affiliations:** 1 Division of Allergy, Pulmonary, and Critical Care Medicine, Department of Medicine, University of Wisconsin School of Medicine and Public Health, Madison, WI, 53792, USA; 2 Department of Medicine, William S. Middleton Veterans Hospital, Madison, WI, 53705, USA

**Keywords:** Thymic stromal lymphopoietin, Eosinophils, Allergic inflammation, Eosinophil derived neurotoxin

## Abstract

**Background:**

Thymic stromal lymphopoietin (TSLP) and eosinophils are prominent components of allergic inflammation. Therefore, we sought to determine whether TSLP could activate eosinophils, focusing on measuring the regulation of TSLPR expression on eosinophils and degranulation in response to TSLP, as well as other eosinophil activation responses.

**Methods:**

Eosinophil mRNA expression of TSLPR and IL-7Rα was examined by real-time quantitative PCR of human eosinophils treated with TNFα and IL-5 family cytokines, and TSLPR surface expression on eosinophils was analyzed by flow cytometry. Eosinophils were stimulated with TSLP (with and without pre-activation with TNFα and IL-3) and evaluated for release of eosinophil derived neurotoxin (EDN), phosphorylation of STAT5, and survival by trypan blue exclusion. A blocking antibody for TSLPR was used to confirm the specificity of TSLP mediated signaling on eosinophil degranulation.

**Results:**

Eosinophil expression of cell surface TSLPR and TSLPR mRNA was upregulated by stimulation with TNFα and IL-3. TSLP stimulation resulted in release of EDN, phosphorylation of STAT5 as well as promotion of viability and survival. TSLP-stimulated eosinophil degranulation was inhibited by a functional blocking antibody to TSLPR. Pre-activation of eosinophils with TNFα and IL-3 promoted eosinophil degranulation at lower concentrations of TSLP stimulation.

**Conclusions:**

This study demonstrates that eosinophils are activated by TSLP and that eosinophil degranulation in response to TSLP may be enhanced on exposure to cytokines present in allergic inflammation, indicating that the eosinophil has the capacity to participate in TSLP-driven allergic responses.

## Background

Thymic stromal lymphopoietin (TSLP) is a cytokine which plays a key role in allergic diseases such as asthma, atopic dermatitis, allergic rhinitis, nasal polyposis, and chronic allergic keratoconjunctivis [1-5]. TSLP is a member of the hematopoietic cytokine family that includes a number of cytokines important in allergic disease including IL-2, IL-4, IL-7, and IL-13. TSLP binds with high affinity to a heterodimeric receptor consisting of the IL-7 receptor - alpha chain (IL-7Rα) and TSLPR (TSLP receptor also known as cytokine receptor-like 2, CRL2). As a member of the hematopoietin receptor family, signaling through activation of TSLPR results in downstream phosphorylation of Signal Transducers and Activators of Transcription (STAT) proteins including, most commonly, STAT5, but also STATs −1, −3, −4 and −6 depending on the cell type examined [6].

A role for TSLP in allergic diseases was initially attributed to its ability to promote TH2 differentiation through a dendritic cell-mediated pathway [7,8]. Subsequently, however, it has been shown that TSLPR is more broadly expressed by a variety of hematopoietic cells (e.g., T cells, B cells, mast cells, eosinophils) as well as structural cells (e.g., epithelial cells) [9-12]. Regulation of TSLPR expression in these cells has not been well studied. In allergic diseases, these TSLPR-expressing cells exist in a milieu of pro-allergic and pro-inflammatory cytokines and other factors (e.g., allergens, bacterial products, lipid mediators) that have the potential to modulate expression of the TSLPR, yet the biological consequences of enhanced expression have not been well examined [13].

Allergic diseases are typically characterized by eosinophilia, both in local tissues and peripheral circulation. In many cases, eosinophils and their granule-associated basic proteins (e.g., EDN, eosinophil cationic protein) are associated with disease severity, yet specific mechanisms promoting eosinophil degranulation are not completely understood [14].

Eosinophils have been shown to express IL-7Rα [2,15]. A recent study reported expression of TSLPR on eosinophils and increased survival and cytokine secretion by TSLP stimulated eosinophils [11]. Here we present the novel findings that TSLP stimulation of eosinophils can also promote TSLPR dependent and TSLP dose-dependent release of EDN as well as phosphorylation of STAT5. Furthermore, we demonstrate that activation of eosinophils with cytokines present in allergic inflammation (TNFα and IL-3) upregulate TSLPR gene expression (but not IL-7Rα) in a dose dependent manner. This upregulation corresponds to increased eosinophil surface expression of TSLPR and enhanced sensitivity to TSLP mediated degranulation.

## Materials and methods

### Human subjects

Peripheral blood was obtained from normal or allergic and/or asthmatic donors ranging in age from 18 to 55 years. Informed consent was obtained before participation and the study was approved by the University of Wisconsin Health Sciences Institutional Review Board, Protocol Number H 2008–0096.

### Cell purifications

Eosinophils were isolated from heparinized blood using magnetic bead negative selection, as described previously [16]. Briefly, blood was separated by density centrifugation (Percoll, 1.090 g/ml) to obtain peripheral blood mononuclear cells (PBMC) and granulocytes. The granulocytes were resuspended and incubated with anti-CD16, anti-CD14, and anti-CD3 magnetic beads (Miltenyi Biotec, Auburn, CA). Negative selection was performed using an AutoMACS® separator (Miltenyi Biotec). The resulting eosinophils were >99% pure and >97% viable. CD3+ T cells were purified from PBMC using Miltenyi Pan T cell Isolation Kit II (Miltenyi Biotec).

### Analysis of TSLPR and IL-7Rα gene expression by quantitative real-time PCR

Purified eosinophils (1 × 10^6^/ml) were incubated with either media alone or TNFα and/or IL-3 (10 ng/ml each; R&D Systems, Minneapolis, MN) for 24 h at 37°C. Total RNA was extracted from 1 × 10^6^ eosinophils using the RNeasy mini kit (Qiagen, Valencia, CA) and reverse transcribed using 400 U of Super Script III reverse transcriptase (Invitrogen, Carlsbad, CA) for 60 min at 37°C in the presence of random hexamer primers (Promega, Madison WI). Real-time quantitative PCR was performed in the Applied Biosystems 7500 sequence detector using human TSLPR-specific primers (CRLF2; Applied Biosystems, Foster City, CA) or human IL-7Rα specific primers (IL-7R; Applied Biosystems) and TaqMan probes (Applied Biosystems). Based on its similar transcription efficiency to the receptor target gene and its consistent expression among treatment groups, the reference gene, β-glucuronidase (GUS) was chosen to normalize the samples. The efficiency of the Taqman assay was determined by assaying serial 10-fold dilutions of target cDNA. All samples were amplified in duplicate, and the mean was obtained for further calculations. The data are expressed as fold inductions using the comparative cycle threshold (Ct) method in which ΔCt = Ct of the chemokine gene minus Ct of GUS; ΔΔCt = ΔCt of activated cells at the indicated time points minus ΔCt of untreated cells. In one subject, the IL-7Rα mRNA quantitative real time PCR did not meet our minimum cycle threshold value criterion and was excluded from our data analysis.

### Flow cytometry

Purified eosinophils (1 × 10^6^/ml) were pre-activated with TNFα and IL-3 (10 ng/ml each) or media alone for 16 h at 37°C. CD3+ T cells were activated with anti-CD3/CD28 beads (Dynabeads, Invitrogen) for 72 h at 37°C. Prior to staining, cells were resuspended in HBSS-BAP (Hanks Buffered Salt Solution, 1 g/L bovine serum albumin, 0.5 g/L sodium azide, and 18 mg/L phenylmethyl sulfonyl fluoride) and non-specific binding of IgG was blocked with normal goat IgG, followed by neutralization of endogenous biotin with streptavidin (Fisher Scientific, Pittsburgh, PA). After staining with goat anti-TSLPR (R&D Systems), a biotinylated rabbit anti-goat IgG secondary antibody (Jackson ImmunoResearch Laboratories, Inc., West Grove, PA) was used and detected using streptavidin conjugated to phycoerythrin (PE; Fisher Scientific). Propidium iodide was added to each tube to determine viability. Detection of phospho-STAT5 was performed using intracellular flow cytometric analysis. Eosinophils were incubated with TSLP (0.5–1.0 μg/ml; R&D Systems), IL-5 (1 ng/ml), or GM-CSF (1 ng/ml) for 15 min, fixed with 2% paraformaldehyde, permeabilized with 90% methanol, and stained with anti-phospho-STAT5-PE (Tyr −^694^, clone 47; BD Biosciences, San Jose, CA) or mouse IgG isotype control. Data was acquired using a FACSCalibur^™^ (BD Bio-sciences) based on gating of viable cells only (except when cells were fixed for intracellular analysis; 10,000 cells counted/tube) and was analyzed using WinMDI (Scripps Research Institute, La Jolla, CA).

### Eosinophil degranulation assay

Eosinophils (1 × 10^6^/ml) were incubated with either TSLP (0.2–2.0 μg/ml), FMLP (10^−7^ M), IL-5 (1 ng/ml) or buffer on 96-well tissue culture plates in 200 μl of HBSS + 0.03% gelatin/well for 4 h at 37°C. In blocking experiments, eosinophils were pre-incubated with goat anti-TSLPR antibody (2.5 μg/ml; AF981, R&D Systems) or goat IgG control prior to treatment as above. In the pre-activation assays, eosinophils were incubated overnight at 37°C with a combination of TNFα and IL-3 (0.1 and 1 ng/ml, respectively) and washed prior to challenge with TSLP. Non-preactivated cells from the same donor were challenged separately for TSLP-stimulated EDN release as described above on the day of purification. Total EDN values were determined by lysis of eosinophils with 0.1% Triton X-100. EDN was measured by ELISA (MBL International, Woburn, MA).

### Eosinophil survival assay

Purified eosinophils (1–1.5 × 10^6^/ml) were suspended in RPMI 1640 supplemented with 5% Fetal Bovine Serum, 100 U/ml penicillin, 100 μg/ml streptomycin, 2 mM glutamine, and 10 mM HEPES and incubated with either IL-5 (0.1 ng/ml), TSLP (0.0625–1 μg/ml) or media for 48 h at 37°C. Viability was evaluated using trypan blue exclusion and the cells were counted using a hemacytometer. Percent survival was determined by dividing the number of viable cells at a given incubation time by the original number of viable cells placed into the well at time zero. Percent viability was determined by dividing the number of viable cells at a given incubation time by the total number of cells (alive + dead) in the well at the given time [17].

### Eosinophil superoxide anion production assay

Purified eosinophils (1 × 10^6^/ml) were pre-activated with TNFα and IL-3 (10 ng/ml each; R&D Systems) or media alone for 24 h at 37°C. Eosinophils were washed and resuspended in HBSS with 0.1% gelatin, incubated at 37°C with 1.2 mg/mL ferro-cytochrome C (Sigma; St. Louis, MO) and stimulated with either buffer, 1 μg/mL TSLP, 1μM FMLP or 1 ng/ml phorbol myristate acetate (PMA, Sigma), as previously described [18]. Superoxide anion production was monitored for 1 h as a colorimetric change at 550 nm. The superoxide production was reported as nmol of cytochrome C per 1.0 × 10^6^ cells, calculated as previously described [18].

### Eosinophil chemotaxis

Purified eosinophils (1 × 10^6^/ml) were pre-activated with TNFα and IL-3 (10 ng/ml each; R&D Systems) or media alone for 24 h at 37°C. Eosinophils were washed and resuspended in HBSS with 0.1% gelatin and cultured in the upper compartment of 24-well 5.0 μm Transwell plates (Costar; Cambridge, MA) as previously described [19]. The bottom compartment contained HBSS with 0.1% gelatin alone or supplemented with either 100 ng/ml eotaxin (Biosource) or 1 μg/ml TSLP. After incubation at 37°C for 1 h, cells were counted in the bottom chamber and the percentage of migration calculated as the number of cells in the bottom chamber divided by the initial number added to the upper chamber.

### Statistical analyses

Paired t-tests were performed for pre-planned comparisons to generate two tailed P values using SigmaPlot (Systat Software, Inc.). A probability of less than 0.05 was considered statistically significant. Cytokine synergistic interaction was evaluated by comparing the calculated additivity (response to TNFα alone + response to IL-3 alone) with the experimental additivity (response to simultaneous stimulation with TNFα and IL-3) by a paired *t*-test analysis. Statistics on mRNA data were performed after log transformation.

## Results

### TSLP activation of eosinophils

**Figure 1:**
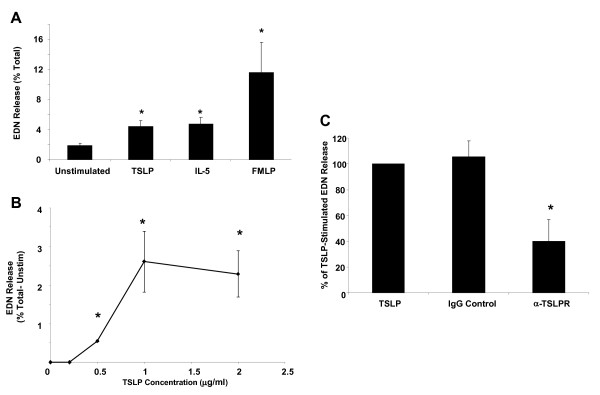
**TSLP stimulates eosinophil degranulation, as measured by EDN release, through a TSLPR specific interaction.** (**A**) Stimulation of eosinophil EDN release, as a percent of total EDN, by TSLP (1 μg/ml) is compared to IL-5 (1 ng/ml) and FMLP (10^−7^ M) stimulated release (n = 6). (**B**) The dose response curve of EDN release, as a percent of total EDN minus unstimulated release, from stimulation with TSLP (0 μg/ml, n = 4; 0.2 μg/ml, n = 3; 0.5 μg/ml, n = 3; 1.0 μg/ml, n = 4; 2.0 μg/ml, n = 3). (**C**) A functional blocking antibody to TSLPR or isotype control (2.5 μg/ml) was incubated with eosinophils prior to stimulation with TSLP. The data are expressed as the percent of TSLP stimulated EDN release (n = 3). *Statistically different from unstimulated (p < 0.05).

Given the role of eosinophil granule proteins in the pathophysiology of allergic diseases, we investigated the ability of TSLP to promote eosinophil degranulation. Eosinophils were incubated for 4 h with various concentrations of TSLP (0.2–2 μg/ml) and with IL-5 (10 ng/ml) and FMLP (10^−7^ M) as positive controls. Stimulation of eosinophils with TSLP (1 μg/ml) significantly increased release of EDN relative to unstimulated eosinophils (Figure 1A, 4.4 ± 0.8% vs. 1.9 ± 0.3%, p = 0.03). This amount of EDN release was comparable to stimulation with 10 ng/ml of IL-5 (4.8 ± 0.8% vs. 4.4 ± 0.8%). TSLP stimulated release of EDN was dose dependent (Figure 1B). No significant increase in EDN release, compared to untreated eosinophils, occurred until 0.5 μg/ml of TSLP was used (p = 0.02). EDN release plateaued at 4.4% with 1.0–2.0 μg/ml TSLP concentrations. A blocking antibody to TSLPR resulted in a 59.9% reduction in TSLP stimulated EDN release (p = 0.05), while an isotype control IgG antibody had no effect (Figure 1C). This confirmed the specificity of the TSLP interaction with TSLPR on eosinophils despite the high concentration of TSLP.

**Figure 2:**
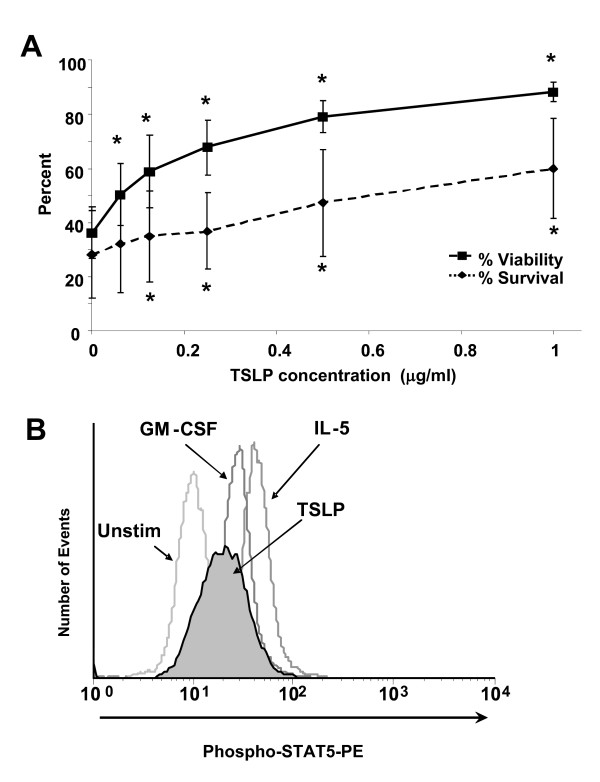
**Effect of TSLP on eosinophil survival and phosphorylation of STAT5.** (**A**) The dose response curve of the effect of TSLP on eosinophil survival, shown as percent survival (0–1 μg/ml, n = 4). (**B**) Effect of TSLPR on eosinophil STAT5 phosphorylation. Histogram of intracellular flow cytometric analysis of phosphotyrosine STAT5 in eosinophils cultured for 15 min in medium (lightest grey line) or TSLP (1 μg/ml, black line with grey shading), compared to stimulation with IL-5 (10 ng/ml, medium grey line) and GM-CSF (10 ng/ml, dark grey line). Data are representative of eosinophils from 3 subjects. The histograms are normalized to number of events. *Statistically different from unstimulated (p < 0.05).

Stimulation with TSLP promoted both eosinophil viability and survival. As shown in Figure 2A*,* TSLP stimulation for 48 h resulted in significantly enhanced viability at concentrations of 62.5 ng/ml and above (p < 0.05) and enhanced survival at 125 ng/ml and above (p < 0.05). IL-5 stimulation (10 ng/mL) was used as a positive control (84.6 ± 5.3% and 86.4 ± 2.6%, p = 0.002 for 48 h).

In eosinophils, STAT5 activation has been shown to enhance survival [20]. In other cell types, such as T cells and mast cells, TSLP mediates STAT5 activation [7,8]. To examine this pathway of TSLPR signaling in eosinophils, we used flow cytometry for detection of phosphorylated STAT5. In Figure 2B, phosphorylated STAT5 was observed with stimulation by 1 μg/ml TSLP (ΔMFI = 10, range = 9 ± 3), 10 ng/ml IL-5 (ΔMFI = 30.6) and 10 ng/ml GM-CSF (ΔMFI = 17.4) compared to unstimulated cells. Some phosphorylation of STAT5 was also detected in response to 0.5 μg/ml TSLP stimulation (ΔMFI = 3 ± 1, histogram not shown).

### Eosinophil expression of TSLPR (mRNA and protein): effect of cytokine pre-activation

**Figure 3:**
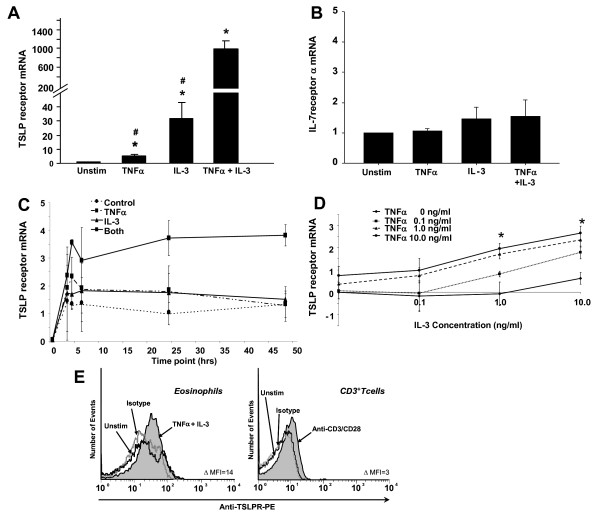
**Eosinophil expression of TSLPR and IL-7Rα.** Quantitative real-time PCR for expression of mRNA for TSLPR (**A**) or IL-7Rα (**B**) was evaluated from eosinophils either untreated or activated for 24 h with TNFα and IL-3, alone and in combination (10 ng/ml). *Statistically different from media control (n = 5, p < 0.05). ^#^ Statistically different from IL-3 combined with TNFα (p < 0.05). (**C**) Time course of expression of mRNA for TSLPR in response to activation with TNFα and/or IL-3 (10 ng/ml) compared to unstimulated control was examined. (**D**) Dose response curve of expression of mRNA for TSLPR in response to TNFα and IL-3 (Y axis) at 24 h *Statistically different from either cytokine alone. (**E**) Representative histograms from flow cytometry of TSLPR on eosinophils (left histogram) treated for 24 h with either media (black line) or a combination of TNFα/IL-3 (10 ng/ml, black line with grey shading). Normal goat IgG was used as an isotype control (grey line). CD3+ T cells stimulated for 72 h with anti-CD3/CD28 coated beads were used as a positive control (right histogram). Data are representative of eosinophils from 3 subjects. The histograms are normalized to number of events. MFI differences are between unstimulated cells and TNFα/IL-3 activated cells.

We sought to determine whether upregulation of TSLPR might enhance activation and decrease the concentration of TSLP required. Expression of TSLPR mRNA was examined in both untreated and activated eosinophils. For activation of eosinophils, we focused on cytokines that are typically expressed in allergic inflammation including the proinflammatory cytokine, TNFα, and the IL-5 family cytokine, IL-3 (alone and in combination). The results of the quantitative real-time PCR are shown in Figure 3A. Expression of TSLPR mRNA was low, but detectable, in untreated eosinophils; however, both cytokines increased expression of TSLPR within 24 h, with greater increases from a combination of TNFα and IL-3. The mRNA expression of TSLPR was induced 5-fold by TNFα (p < 0.001) and 32-fold by IL-3 (p = 0.002); however, the combination of TNFα and IL-3 induced a significant synergistic increase of 991-fold (p < 0.001). Since the TSLP functional receptor consists of a heterodimeric complex of TSLPR and IL-7Rα, we also examined the eosinophil mRNA expression of IL-7Rα (Figure 3B). Expression of IL-7Rα was detectable, but did not vary significantly with any of the cytokine treatments.

Time course (3–48 h) and dose response (0.1–10 ng/ml) experiments were also conducted (Figures 3C & D respectively). The time course experiments showed that pre-activation with TNFα and IL-3 (10 ng/ml) resulted in increased TSLPR mRNA expression that peaked at 4 h and remained elevated through 48 h. Dose response curves showed that this increased expression was dose dependent and that the combination of TNFα and IL-3 was more potent than either cytokine alone (p < 0.05 at 1 and 10 ng/ml).

As unstimulated eosinophils had low surface expression of TSLPR and the combination of TNFα and IL-3 significantly upregulated TSLPR mRNA, we examined surface TSLPR protein expression following overnight incubation with these cytokines (10 ng/ml). CD3+ T cells were incubated for 72 h with anti-CD3/CD28 coated beads, previously shown to upregulate TSLPR on T cells, as a positive control [9]. In Figure 3E, histograms show a shift in mean fluorescence intensity of TNFα/IL-3 activated eosinophil surface staining for TSLPR compared to unstimulated eosinophils (ΔMFI = 14, range = 6–14; left histogram) and the shift in mean fluorescence of ant-CD3/CD28 activated CD3+ T cells compared to untreated T cells (ΔMFI = 3, right histogram).

### Effect of cytokine-mediated pre-activation on TSLP-stimulated eosinophil function

**Figure 4:**
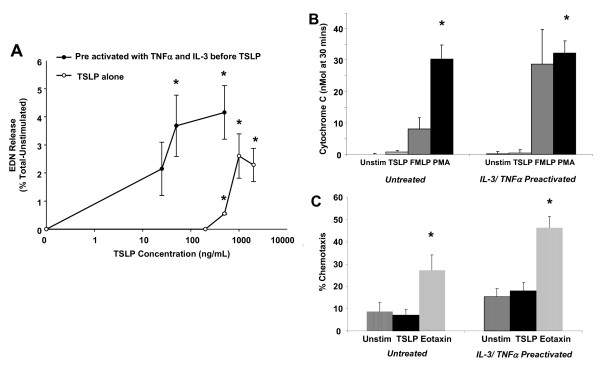
**Effect of pre-activation with TNFα and IL-3 on TSLP-mediated eosinophil activation.** (**A**) The dose response curve of EDN release (as a percent of total EDN minus unstimulated release) in response to stimulation with TSLP (25–500 ng/ml, n = 3), following pre-activation for 24 h with TNFα and IL-3 (0.1 ng/ml and 1 ng/ml respectively, filled circles) is compared to EDN release from TSLP stimulated eosinophils (250–1,000 ng/ml) in the absence of pre-activation (open circles, n = 3–4). *Statistically different from unstimulated (p < 0.05). (**B**) Superoxide production expressed as nmol cytochrome C in response to TSLP (1 μg/ml) is compared to unstimulated control and positive controls (FMLP, PMA) with and without pre-activation with TNFα/IL-3 (10 ng/ml; n = 3). (**C**) Percent chemotaxis in response to TSLP (1 μg/ml) with and without pre-activation with TNFα/IL-3 (10 ng/ml) is compared to unstimulated control and eotaxin positive control (100 ng/ml, n = 3). *Statistically different from unstimulated (p < 0.05).

Since stimulation with TNFα and IL-3 upregulated TSLPR, we examined whether pre-activation would decrease the threshold for TSLP mediated degranulation. As TNFα and IL-3 activation of eosinophils also causes dose dependent EDN release (data not shown) we used the lowest concentrations of TNFα and IL-3 that resulted in upregulation of TSLP mRNA while minimizing background EDN release. Figure 4A shows that pre-activation of eosinophils with TNFα (0.1 ng/ml) and IL-3 (1.0 ng/ml) resulted in enhanced sensitivity to TSLP-mediated degranulation at lower concentrations of TSLP (50–500 ng/ml tested) which was statistically significant compared to unstimulated eosinophils at 100 and 500 ng/ml of TSLP (p = 0.05). As shown in Figure 4B and C, TSLP did not promote either superoxide production or chemotaxis even with TNFα and IL-3 pre-activation. We were unable to evaluate the effect of pre-activation with TNFα and IL-3 on TSLP-stimulated survival and STAT5 phosphorylation because of the direct effect of IL-3 on these functions.

## Discussion

We have demonstrated for the first time that eosinophils respond directly to TSLP with degranulation (release of EDN) and activation of STAT5. Furthermore, we have shown that eosinophil expression of both TSLPR mRNA and surface protein can be markedly upregulated by a combination of TNFα and IL-3, cytokines present in allergic inflammation. More importantly, we went on to demonstrate that upregulation of eosinophil surface expression of TSLPR significantly enhanced sensitivity of eosinophils to TSLP-mediated degranulation. While we were also interested in whether TNFα and IL-3 pre-activation would enhance TSLP activation of eosinophil survival and STAT5 phosphorylation, we were unable to evaluate these functions as IL-3 stimulation of eosinophils promotes both, survival and STAT5 phosphorylation [21].

Interestingly, while TNFα and IL-3 had a profound effect on upregulation of TSLPR mRNA, they did very little to modulate expression of mRNA for IL-7Rα. This suggests that, while both the TSLPR and IL-7Rα are required for high affinity binding and signaling via TSLP, TSLPR could be regulated independently from IL-7Rα resulting in expression of TSLPR that is not coupled with the heterodimeric complex required for signaling.

It is notable that the concentration of TSLP (0.5–1.0 μg/ml) required for activation of eosinophil degranulation (in the absence of pre-activation) is greater than the concentrations of GM-CSF and IL-5 (10 ng/ml) required to promote comparable degranulation responses. However, the activation of the IL-7 receptor complex on eosinophils (which shares IL-7Rα with the TSLPR) similarly required high concentrations of IL-7, 50 nM or 0.9 μg/ml [15]. One possible explanation is that additional factors present in the *in vivo* microenvironment during allergic inflammation prime eosinophils to respond to lower concentrations of TSLP. This is supported by our observation that pre-activation of eosinophils with TNFα and IL-3 enhances both TSLPR expression and sensitivity to TSLP stimulation *in vitro*, suggesting that TSLP responses may be reserved for eosinophils in an inflammatory environment. Another possibility is that eosinophil responses to TSLP are biologically programmed to be more conserved and less promiscuous, reserved for responses to a bolus of TSLP released directly to tissue eosinophils in the activated epithelium.

In asthma and allergic diseases, eosinophil recruitment, survival, and cytotoxic effector functions are largely regulated by IL-5 family cytokines (IL-3, IL-5, GM-CSF). Besides direct effects on eosinophil activation, these cytokines enhance eosinophil responsiveness to second stimuli, such as chemokines and FMLP [22]. The mechanism for priming involves activation of signaling molecules such as Lyn and ERK1/2. Studies have shown that *in vitro* priming of blood eosinophils with IL-5 family cytokines can result in eosinophils with a similar phenotype to airway eosinophils [23]. Our studies demonstrate a comparable priming effect of TNFα and IL-3 for TSLP mediated eosinophil degranulation. The TNFα and IL-3 pre-activation was shown to increase TSLPR mRNA and surface expression. However, it is possible that other intracellular signaling events are also primed to facilitate TSLP mediated activation of eosinophils.

Our findings differ from a recently published study, using lower concentrations of TSLP (and ECP as a measure of degranulation) which reported that stimulation of eosinophils with TSLP was unable to promote eosinophil degranulation and phosphorylation of STAT5 [11]. These discrepancies could be explained by differences between our approaches, including concentrations of TSLP tested, as well as differences in experimental protocols such as the degranulation and phospho-STAT5 detection. EDN is a more sensitive measure of eosinophil degranulation than ECP because it has a higher soluble recovery from lysed cells [24]. However, our study supports the finding of Wong, et al. that TSLP can promote viability (as well as survival) at lower concentrations than are required for degranulation. Based on the literature, promotion of eosinophil survival by IL-5 family cytokines occurs through a pathway involving either STAT5 or STAT3 phosphorylation [20]. Studies in T cells and mast cells have demonstrated that TSLPR activation results in STAT5 phosphorylation [7,8]. Although Wong, et al. did not detect STAT5 phosphorylation by TSLP in eosinophils using Western blot, we utilized flow cytometry to detect phospho-STAT5 in eosinophils which may be a more sensitive technique and is more consistent with the findings in T cells and mast cells.

Studies using various genetically deficient mice have established the requirement for eosinophils in both local skin and systemic inflammatory responses to intradermally administered TSLP [25]. These studies demonstrated that T cells and eosinophils were required, whereas mast cells and TNF-α were dispensable. In a separate study, mice with keratinocyte- or lung-specific overexpression of TSLP were shown to have an atopic dermatitis- or asthma-like phenotype, characterized by eosinophilia, while mice lacking the TSLP receptor have considerably attenuated disease, lacking eosinophilia [26]. In a human study, focusing on nasal polyposis, it was reported that high expression of TSLP in nasal polyps strongly correlated to eosinophils and IgE suggesting a potential role for TSLP in the pathogenesis of nasal polyps by regulating Th2 type and eosinophilic inflammation [4]. While these studies establish a correlation between TSLP and eosinophils, they fall short of establishing a direct link between eosinophil activation and TSLP.

## Conclusion

Our studies demonstrate that TSLP, previously thought to preferentially target dendritic cell and T cell interactions, can also promote eosinophil activation and degranulation, and that eosinophil activation in response to TSLP may be enhanced on exposure to cytokines present in allergic inflammation. It is difficult to predict the role of TSLP activation of eosinophils in allergic diseases, when so many other factors are present that could similarly promote eosinophil degranulation and activation. Activation of eosinophils via receptors for cytokines, immunoglobulins, and complement can lead to the secretion of an array of proinflammatory cytokines, growth factors, chemokines and lipid mediators. Rather than TSLP being singularly important in eosinophil activation, TSLP may be involved in altering eosinophil responses in the presence of other activating influences. However, it is clear from our data and other recent studies that the eosinophil has the capacity to participate in TSLP-driven allergic responses.

## Abbreviations

CD, Cluster of differentiation; CRLF2, Cytokine receptor-like factor 2; Ct, Cycle threshold; EDN, Eosinophil derived neurotoxin; ELISA, Enzyme-linked immunosorbent assay; ERK, Extracellular-signal-regulated kinase; FMLP, N-Formylmethionyl-leucyl-phenylalanine; GM-CSF, Granulocyte-macrophage colony-stimulating factor; GUS, β-glucuronidase; HBSS, Hanks buffered salt solution; HBSS-BAP, Hanks buffered salt solution 1 g/L bovine serum albumin, 0.5 g/L sodium azide, and 18 mg/L phenylmethyl sulfonyl fluoride; HEPES, 4-(2-hydroxyethyl)-1-piperazineethanesulfonic acid; Ig, Immunoglobulin; IL, Interleukin; IL-7Rα, Interleukin 7 receptor alpha; Lyn, Member of the Src family of protein tyrosine kinases; MFI, Mean fluorescence intensity; mRNA, Messenger ribonucleic acid; PBMC, Peripheral blood mononuclear cells; PCR, Polymerase chain reaction; PE, Phycoerythrin; PMA, Phorbol myristate acetate; RPMI, Roswell park memorial institute medium; STAT, Signal transducers and activators of transcription; TNFα, Tumor necrosis factor alpha; TSLP, Thymic stromal lymphopoietin; TSLPR, Thymic stromal lymphopoietin receptor.

## Competing interests

The authors declare that there are no competing interests.

## Authors’ contributions

EBC conceived of the study, participated in its design, carried out flow cytometry and eosinophil functional studies and analyzed results. JLS participated in the study design, coordination, carried out eosinophil functional studies, and formatted the final version of the manuscript and figures for submission. EAS, KEF carried out RNA and eosinophil functional studies, and analyzed results. SKM conceived of the study and participated in its design. All authors were involved in drafting or critically revising the manuscript and approved the final version.
